# Evaluating the Effectiveness of Biodiverse Green Schoolyards on Child BMI z-Score and Physical Metrics: A Pilot Quasi-Experimental Study

**DOI:** 10.3390/children12070944

**Published:** 2025-07-17

**Authors:** Bo H. W. van Engelen, Lore Verheyen, Bjorn Winkens, Michelle Plusquin, Onno C. P. van Schayck

**Affiliations:** 1 Department of Family Medicine, Care and Public Health Research Institute (CAPHRI), Maastricht University, P.O. Box 616, 6200 MD Maastricht, The Netherlands; onno.vanschayck@maastrichtuniversity.nl; 2 Centre for Environmental Sciences, Hasselt University, 3590 Hasselt, Belgium; lore.verheyen@uhasselt.be (L.V.); michelle.plusquin@uhasselt.be (M.P.); 3 Department of Methodology and Statistics, Care and Public Health Research Institute (CAPHRI), Maastricht University, P.O. Box 616, 6200 MD Maastricht, The Netherlands; bjorn.winkens@maastrichtuniversity.nl

**Keywords:** childhood obesity, green schoolyards, anthropometric measures, BMI z-score, school-based intervention

## Abstract

**Background:** Childhood obesity is a significant public health issue linked to poor diet, low physical activity, and limited access to supportive environments. Green schoolyards may promote physical activity and improve health outcomes. This study evaluated the impact of the Green Healthy Primary School of the Future (GHPSF) intervention—greening schoolyards—on children’s BMI z-scores, waist circumference, and hip circumference over 18 months, and compared these effects to those observed in the earlier Healthy Primary School of the Future (HPSF) initiative. **Methods:** This longitudinal quasi-experimental study included two intervention and two control schools in Limburg, a province both in the Netherlands and Belgium. Children aged 8–12 years (n = 159) were assessed at baseline, 12 months, and 18 months for anthropometric outcomes. Linear mixed models were used to estimate intervention effects over time, adjusting for sex, age, country, and socioeconomic background. Standardized effect sizes (ESs) were calculated. **Results:** The intervention group showed a greater reduction in BMI z-scores at 12 months (ES = −0.15, *p* = 0.084), though this was not statistically significant. Waist circumference increased in both groups, but less so in the intervention group, at 12 months (ES = −0.23, *p* = 0.057) and 18 months (ES = −0.13, *p* = 0.235). Hip circumference and waist–hip ratio changes were minimal and non-significant. GHPSF effect sizes were comparable to or greater than those from the HPSF initiative. **Conclusions:** Though not statistically significant, trends suggest that greening schoolyards may support favorable changes in anthropometric outcomes. Further research with larger samples and longer follow-up is recommended.

## 1. Introduction

Worldwide, obesity is a major health concern [1]. Globally, over 390 million children and adolescents aged 5–19 were overweight in 2022, of whom 160 million were classified as obese [2]. In 2021, 11.9% of the 4–12-year-old Dutch children were overweight, and 3.6% were obese [3]. Compared to the previous year, there was a notable increase of 2.3% in the total prevalence of overweight and obesity among this age group [3]. These numbers are concerning since obesity increases the risk of developing cardiovascular disease. In addition, obesity is also associated with several other health problems, such as type 2 diabetes and cancer [4,5].

Engaging in an unhealthy lifestyle, characterized by insufficient physical activity and unhealthy eating behaviors, is associated with an increased risk of being overweight and obesity [4]. The World Health Organization recommends that children and adolescents aged 5–17 years engage in an average of at least 60 min per day of moderate-to-vigorous intensity aerobic physical activity—such as brisk walking, cycling, or active play—while also incorporating vigorous-intensity activities like running or competitive sports, and muscle- and bone-strengthening exercises such as jumping, climbing, or bodyweight resistance training at least three times per week to support cardiorespiratory and musculoskeletal health [5]. Despite these recommendations, global estimates indicate that over 80% of adolescents aged 5–17 years fail to meet these physical activity guidelines [6]. Moreover, nutritional behavior was unsatisfactory, with only 50.9% of the Dutch children in primary school consuming the recommended two servings of fruit per day and merely 46.2% meeting the target of 150 g of daily vegetable intake [7].

Despite numerous school-based interventions targeting childhood obesity, long-term effectiveness remains mixed, particularly in low-SES settings. Recent advances in quasi-experimental research designs have improved causal inference in real-world settings where randomization is often impractical [8,9]. These studies highlight the importance of integrating environmental changes, such as schoolyard modifications, into health promotion strategies.

Unhealthy habits are often formed at a young age already and carried out throughout one’s life. For this reason, it is important to promote healthy behavior early on in life [10]. Encouraging healthy habits in early childhood can have a positive impact on both children’s health and their academic success, potentially leading to better long-term health outcomes [11].

Schools have been considered important environments for promoting healthy habits in early childhood for several reasons. First, schools have the capacity to reach all children from different backgrounds. The structured school environment enhances the ease of implementing interventions for children of different social economic statuses. Additionally, children spend a significant portion of their day at school, often consuming one to two meals there. Schools also provide opportunities for physical activities and health education [12,13]. By providing long-lasting exposure to elements of a healthy lifestyle in school, unhealthy behaviors may be modified [5,14]. Given the real-world constraints of implementing interventions at the school level, quasi-experimental designs are increasingly used to evaluate the effectiveness of school-based health initiatives without requiring random assignment [14,15].

This led to the idea of the Healthy Primary School of the Future (HPSF): a school-based intervention implemented in Limburg, a southern province of the Netherlands, aimed at improving dietary habits and physical activity [8]. The full intervention (HPSF) included the provision of daily lunches and the incorporation of structured physical activity sessions following lunch [11,14].

Building upon this, the Green Healthy Primary School of the Future (GHPSF) initiative extends the HPSF by focusing on greening schoolyards to enhance physical activity. Green spaces have been shown to support increased physical activity and psychological well-being, and to potentially lower obesity risks [15,16,17]. However, limited empirical evidence exists on whether biodiverse green schoolyards can lead to measurable improvements in obesity-related metrics when compared with traditional school environments. It is hypothesized that greening the schoolyard will have a comparable effect on the BMI z-score to that which the HPSF would have, namely a decreasing trend.

The main objective of the current explorative study is to assess the effect after a one-and-a-half-year follow-up of the implementation of a more biodiverse, green schoolyard renovation on children’s BMI z-score, waist circumference, and hip circumference compared with those of children in the control schools without a schoolyard renovation. A secondary objective is to compare these results with the earlier obtained results of the HPSF study to see if the observed changes are in the same direction and of the same magnitude [8].

## 2. Materials and Methods

### 2.1. Study Design

The current study had a longitudinal quasi-experimental design, involving two intervention schools and two control schools. Building on the considerable experience with the Healthy Primary School of the Future (HPSF) in the Netherlands [14,17] and inspired by successful initiatives in both the Netherlands and Belgium, two provinces—each on one side of the border—jointly decided to initiate an explorative study. This led to the development of the integrated initiative Green Healthy Primary Schools of the Future (GHPSF), aimed at investigating the potential effects of combining health promotion with greening school environments. One intervention and one control school were located in the province of Limburg in Belgium. The other intervention and control schools were in the province of Limburg in the Netherlands. Measurements were conducted from September to November 2021 (0), 2022 (12 months), and April and May 2023 (18 months). A detailed description of the study is reported in van Engelen et al. [18].

### 2.2. The Green Healthy Primary School of the Future

Two collaborating organizations, Maastricht University and Hasselt University, developed the idea for the Green Healthy Primary School of the Future. A maximum of 4 schools was included in the study. In June 2021, a call was placed to recruit schools who planned on greening their schoolyard in the near future. Two intervention schools were selected based on their possibility of starting the intervention. The greening started directly after the baseline measurement. Before the greening, the playground consisted mainly of concrete bricks and playground equipment. Through the greening process, the following initiatives were introduced to the schoolyards: (i) creation of adventurous and diverse playgrounds, (ii) development of green relaxation areas, (iii) planting of flowers and/or shrubs focused on increasing biodiversity and/or (iv) creation of a variety of activity zones. In contrast, the control schools underwent no change concerning the schoolyard; the schoolyards consisted mainly of paved surfaces and playground equipment. The greening process included the installation of adventurous play zones (e.g., climbing elements, natural mounds), development of shaded green relaxation areas, planting of diverse shrubs and flowers to enhance biodiversity, and creation of defined activity zones to encourage varied physical movement. These features were designed not only to increase physical activity but also to stimulate curiosity, social interaction, and nature engagement.

### 2.3. Study Population

The intervention and control schools are situated in the province of Limburg, a region in the south-eastern part of the Netherlands and the north-eastern side of Belgium. This region is characterized by a low to average socioeconomic status (SES) and exhibits higher prevalence rates of unhealthy lifestyle behaviors and overweight compared to the rest of the Netherlands and Belgium [19,20]. Details on the recruitment process for the participants have been provided in the protocol publication [18]. Children aged 8–12 years were invited to participate in the study. Recruitment was conducted through informational brochures distributed to parents and children. Additionally, the research team visited classrooms to inform children about the study and encourage them to discuss participation with their parents [18].

### 2.4. Measurements

Data collection was conducted during multiple measuring weeks, in which all measurements were conducted during one week for each timepoint (0, 12, 18 months). Inter-rater variability was minimized by training researchers according to a strict protocol [16]. Information on the age, study year, and sex of the participating children was provided by the legal guardian. A digital questionnaire was administered to parents to gather information on the children’s socioeconomic background (SEB). SEB was measured using standardized scores on maternal education level and paternal education level based on the highest completed education in the family (low (primary school); middle (secondary school); high (higher education/university)). All anthropometric measurements were conducted by trained researchers who followed a standardized measurement protocol. Training sessions were held prior to each measurement round to ensure inter-rater reliability and procedural consistency.

### 2.5. BMI z-Score

Height, weight, hip, and waist circumference measurements were conducted in children aged 8–12 years old. Height was measured using a portable stadiometer (Seca 213, Hamburg, Germany), and weight was recorded using a calibrated digital scale (Tanita, Tokyo, Japan). Waist and hip circumference were measured with a flexible, non-stretchable tape at standardized anatomical landmarks. All protocols were based on procedures described in Willeboordse et al. [14]. These anthropometric measurements were integrated into the measuring week. Further details of the measurement protocol can be found in Willeboordse et al. [8]. BMI was determined using height and weight, with overweight and obesity defined according to age- and sex-specific BMI thresholds [21]. BMI z-scores were calculated by using Dutch reference values [22].

### 2.6. Statistical Analyses

Given the exploratory nature of this study, no formal sample size or power calculation was performed. The main goals were to assess the potential impact of the intervention and to examine whether these effects were comparable to those found in the HPSF study [9].

Descriptive statistics are presented for categorical variables as the number of children, with percentages, while the mean with standard deviation (SD) was used for numerical variables. Differences in categorical and numerical variables between the groups were assessed using chi-square or Fisher’s exact tests and independent-samples *t*-tests, respectively.

To assess the difference in longitudinal trends between the intervention and control group, a marginal model for repeated measures was used, where group (intervention/control), time (0, 12, 18 months) and the interaction between group and time were included as fixed factors and an unstructured covariance structure was used for the repeated measures, as the number of repeated measures was limited to three. In addition, variables potentially associated with the outcome and/or missing values were included as fixed factors, including sex (male/female), age (in years), country (Belgium/Netherlands), and highest family education level (highest level of maternal and paternal educational level: low (primary school); middle (secondary school); high (higher education/university)). This model used all available data, assumed missing values to be random (MAR), and provided intervention effects at 12 and 18 months that were corrected for baseline differences in the outcome and the other variables included in the fixed part of the model. For the sensitivity analyses, we performed the same analyses without the highest family education level for all outcomes and without age and sex for the BMI z-score, as these scores were corrected for age and sex.

Standardized effect sizes (ESs) were computed as estimated intervention effects (corrected for the baseline differences as well as for the aforementioned other baseline characteristics) divided by the residual SD at baseline (pooled over both groups).

Data was analyzed using IBM SPSS Statistics for Windows (version 28.0.1.1, Armonk, NY, USA: IBM Corp.), and two-sided *p*-values ≤ 0.05 were considered statistically significant.

## 3. Results

**Figure 1:**
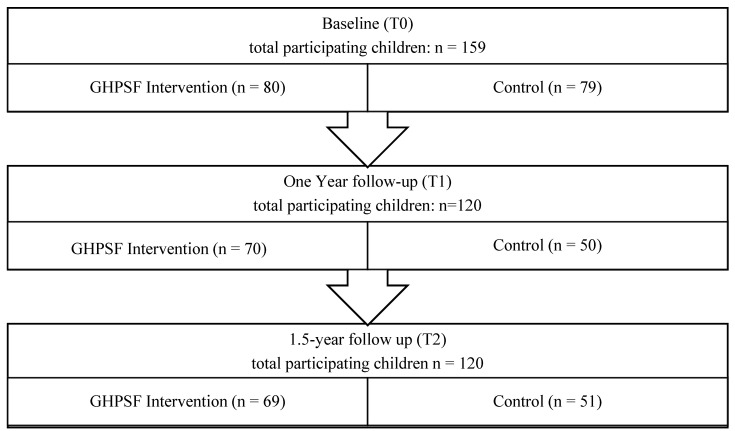
Flow chart of the study course. The reasons for drop-outs were switching to another school and actively halting participation.

Of all the children (n = 482) invited to participate in the study, 159 (33%) joined the study and had at least one BMI z-score measurement during the study. Of these children, 120 (75%) were measured after 1 year and 1.5 years of follow-up (Figure 1). In this study population, 44% of the participating children were boys and 56% were girls‘; their mean age was 10.1 years (Table 1). Their average height was 142.7 cm, and their average weight was 36.3 kg. The mean waist circumference was 62.4 cm, with 76.3 cm as the hip circumference, and with a waist hip ratio of 0.82. The BMI z-scores had an average value of 0.41, and the majority (62%) came from a high social economic background. None of these baseline characteristics differed significantly between the groups (Table 1).

**Figure 2:**
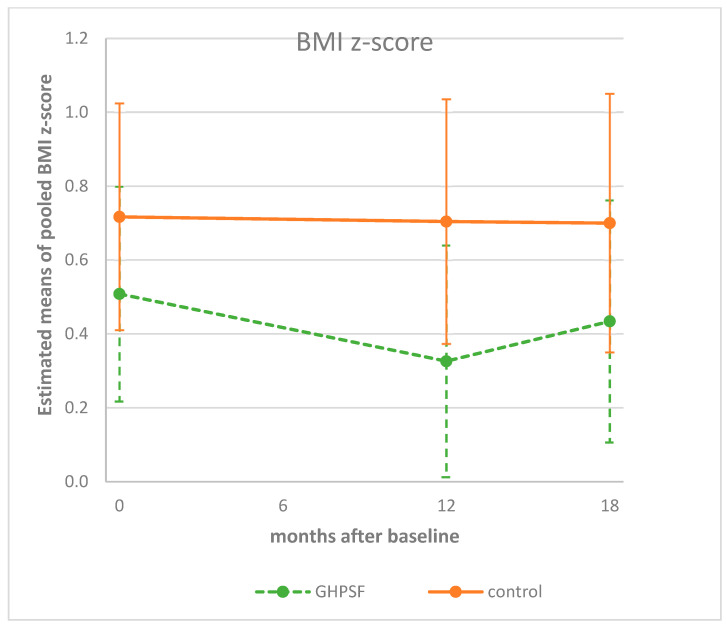
Estimated means (95%CI) of children’s BMI z-scores at baseline, and at 12 and 18 months, corrected for sex, age, country (Belgium/Netherlands), and highest family education level. GHPSF, Green Healthy Primary School of the Future.

Linear mixed-model analyses were conducted to study the differences in longitudinal trends between the two groups. For the BMI z-score, a difference in the decrease from the baseline can be observed after one year of follow-up between the two groups (Table 2). The BMI z-score decreased more in the intervention group (Δz-score: −0.182) compared to the control group (Δz-score: −0.013; Figure 2), although not significantly (*p* = 0.084, ES = −0.15). In addition, after 18 months, the decrease from baseline was still observable, although there was a smaller difference between the groups (Δz-score: −0.074 versus −0.017, *p* = 0.540, ES = −0.05) (Figure 2).

**Figure 3:**
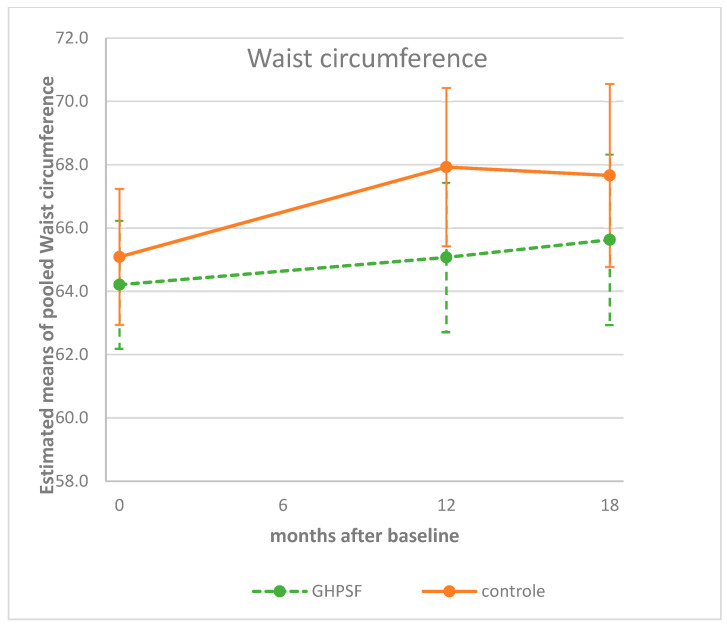
Estimated means (95%CI) of children’s waist circumferences at baseline, and at 12 and 18 months, corrected for sex, age, country (Belgium/Netherlands), and highest family education level. GHPSF, Green Healthy Primary School of the Future.

Waist circumference was not significantly different between the groups after 1 year of follow-up in the intervention group (*p* = 0.057) or at the 18-month follow-up (*p* = 0.235) (Table 2). Estimated data after 12 months show an increase in waist circumference in comparison with the baseline in both groups (Δz-score: 0.861 versus 2.837) (Figure 3). After 18 months, this increase from the baseline was still present in the GHPSF (Δz-score: 1.421 cm) as well as in the control schools (Δz-score: 2.573 cm) (Figure 3).

**Figure 4:**
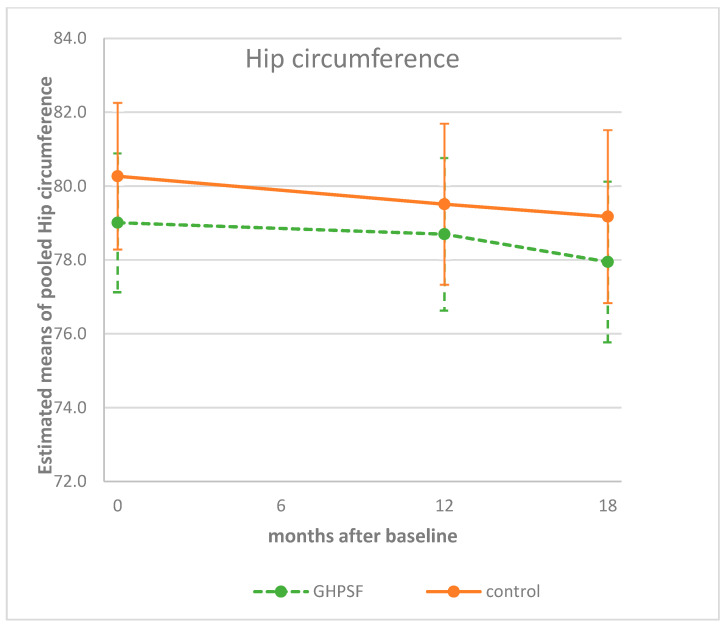
Estimated means (95%CI) of children’s hip circumferences at baseline, and at 12 and 18 months, corrected for sex, age, country (Belgium/Netherlands), and highest family education level. GHPSF, Green Healthy Primary School of the Future.

For hip circumference, changes between groups were minimal. At 12 months, the intervention effect was 0.45 cm (95%CI: −1.10, 2.00; *p* = 0.567), and at 18 months, it was 0.03 cm (95%CI: −1.80, 1.87; *p* = 0.970), indicating no clear difference in hip circumference changes between the intervention and control groups (Figure 4).

**Figure 5:**
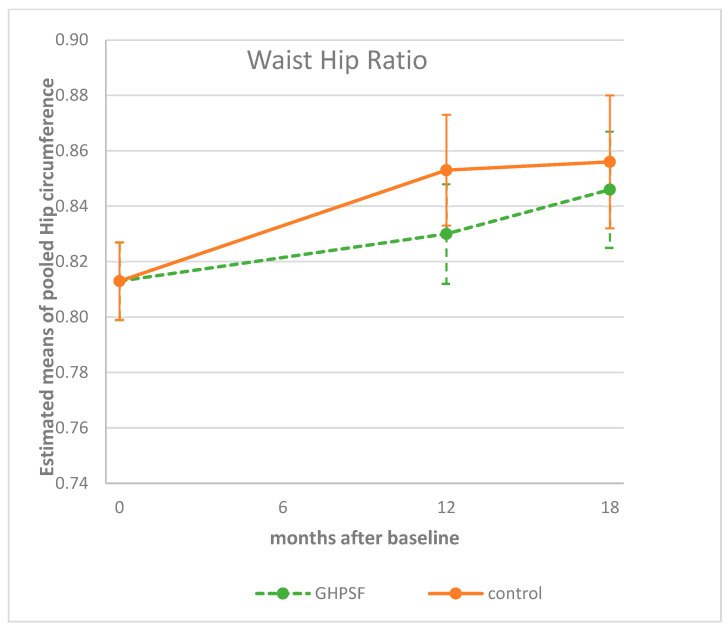
Estimated means (95%CI) of children’s waist hip ratios at baseline, and at 12 and 18 months, corrected for sex, age, country (Belgium/Netherlands), and highest family education level. GHPSF, Green Healthy Primary School of the Future.

At the 12-month follow-up, the waist hip ratio (WHR) increased in both groups. The intervention group (GHPSF) showed a smaller mean increase in estimated mean WHR (from 0.813 to 0.830) compared to the control group (from 0.813 to 0.853). Although this difference suggested a potential effect of the intervention, it did not reach statistical significance despite it reaching a borderline significant association level (Δz-score: 0.017 vs. 0.040, *p* = 0.090; Table 2, Figure 5). By 18 months, both groups experienced further increases in WHR (GHPSF to 0.846; control to 0.856), and the between-group difference remained non-significant (*p* = 0.420; Table 2, Figure 5).

To assess the robustness of the intervention effects, we performed additional subgroup analyses stratified by sex (boys and girls). These exploratory analyses revealed differential patterns in effect sizes between boys and girls (Supplementary Table S2a,b). For the BMI z-score, the intervention effect was more pronounced in boys (ES = −0.20 at 12 months; ES = −0.08 at 18 months) compared to girls (ES = −0.08 and 0.001, respectively). For waist circumference, the treatment effect was stronger in girls (ES = −0.33 at 12 months; −0.25 at 18 months) than in boys (−0.15 and −0.05). For the waist–hip ratio (WHR), a consistently stronger effect was seen in girls (ES = −0.62 at 12 months; −0.58 at 18 months) compared to boys (ES = −0.20 and 0.14). These intervention effects did not reach statistical significance.

## 4. Discussion

The current explorative study looked into the effects of the Green Healthy Primary School of the Future (GHPSF) initiative on children’s BMI z-scores, waist circumference, and hip circumference, comparing these outcomes with those of the control schools. Although the standardized effect size (ES) differences between the intervention and control groups after 12 months were not statistically significant, they were comparable in magnitude to the ES differences observed in the HPSF study, suggesting the potential relevance of the intervention’s impact.

Body mass index (BMI) is expressed as a z-score, which accounts for sex- and age-related variations. In contrast, waist circumference (WC) is not standardized as a z-score and therefore not adjusted for sex and age. Recent research recommends the routine use of waist circumference in both research and clinical practice, given its superior ability to capture visceral adiposity and predict cardiometabolic risk [23]. Excess adiposity should be confirmed by combining BMI with at least one additional validated anthropometric indicator, such as waist circumference or waist hip ratio, using age-, sex-, and ethnicity-appropriate cutoff points [24]. As a result, when BMI was represented as a z-score, a horizontal line was observed in the control group, with a decrease indicating a favorable outcome in the intervention group (Figure 2). As for WC, due to the lack of age correction, one would typically expect an increase, at least in the control group. A smaller (or absence of) increase in WC in the intervention group would therefore indicate a favorable outcome (Figure 3).

Due to limited funding, this study was designed as an exploratory evaluation involving only two intervention and two control schools, and it is therefore likely underpowered. Ideally, data collection would have occurred at multiple follow-up intervals, including at 6 months, to track more granular changes in anthropometric outcomes. However, resource constraints precluded additional timepoints. As such, the study focused on 12- and 18-month measurements to balance feasibility with meaningful longitudinal assessment within a quasi-experimental framework. Nevertheless, the observed effects were comparable to or even greater than those reported in the HPSF study, underscoring the relevance of the intervention. Ideally, data collection would have occurred at 0, 12, and 24 months to capture longer-term trends, but resource constraints restricted follow-up to 18 months.

Furthermore, sex-stratified subgroup analyses revealed notable differences in intervention responses. Boys showed larger reductions in BMI z-score, while girls exhibited greater reductions in waist circumference and a consistent, borderline significant decrease in WHR. Although these results were not statistically significant, they suggest possible sex-specific pathways through which children respond to green schoolyard interventions. This exploratory finding merits further investigation in future studies with larger sample sizes and statistical power to detect interaction effects.

Body mass index z-scores in children have been shown to fluctuate with seasonal patterns, reflecting changes in energy balance associated with physical activity, diet, and environmental factors. Colder seasons may contribute to increased sedentary behavior and less outdoor activity, leading to modest but significant increases in BMI-z, particularly in temperate climates [25]. These seasonal influences may help explain the observed consistent lower increases in BMI z-(and WC) scores at 18 months as compared to 12 months. Although seasonality may influence outcomes, its impact on the current results is expected to be limited due to the use of mixed model analyses, which account for time as a repeated factor. Therefore, the analysis is considered representative of what occurred over the intervention period.

A secondary objective of this study was to compare these results with the earlier obtained results of the HPSF study. The standardized effect size of the current study on the BMI z-score was −0.15 (Table 2, Supplementary Table S1), which is assumed to be a small effect [26]. In the HPSF study, the standardized effect size after 1 year of intervention was only −0.04, and after 4 years, it was −0.17 [8]. Furthermore, the standardized effect size for waist circumference in the current GHPSF study was −0.23, compared to −0.06 after 1 year and −0.22 after 4 years in the full HPSF intervention [8], suggesting that the short-term impact of the GHPSF intervention may be at least comparable to the longer-term effects of HPSF. Even though these effect sizes are assumed to be small, they were observable after only 1 year and they seem to suggest a decreasing trend in BMI-z scores. Given the exploratory nature of this study and the limited number of participating schools, these effects were likely not statistically significant—longer follow-up and/or inclusion of more schools would be needed to confirm them. These results may make up a broader picture that suggests that children increase their active play behavior following a green schoolyard renovation, compared to concrete schoolyards [27].

The GHPSF initiative appears promising in its potential to influence BMI z-scores positively. The observed trends, which align with previous research, demonstrate that green spaces have numerous benefits, particularly for children [16]. These benefits include physical and mental health, cognitive and behavioral development, stress reduction, and social well-being. Access to green spaces is also associated with increased physical activity. Green schoolyards provide more opportunities for active play and exercise, which can lead to an increase in physical activity and a reduction in sedentary time [14]. A green and biodiverse environment can reduce stress and improve overall well-being [28]. A better sense of well-being can lead to healthier behavioral choices, including increased physical activity and healthier eating habits [15]. Greening schoolyards can bridge health equity gaps by providing safe, natural play areas for children, regardless of their socioeconomic background [17].

A longer follow-up period is essential to determine if the observed results are sustainable and not merely a result of the initial enthusiasm and cooperation from children in response to the new school changes, which might cause leap intervention effects to diminish over time. Additional outcomes should also be investigated to assess the full impact of the GHPSF intervention. These include evaluating children’s physical activity behavior, overall well-being, cognitive performance, and the cost-effectiveness of the schoolyard renovations. These outcomes are included in the overall study design and will be investigated further [8].

The quasi-experimental design is a limitation of this study, as we were unable to randomize schools, and—like in any school-based intervention—blinding of participants was not possible. An advantage of the current intervention study, with its inclusion of a follow-up until 18 months, is that it allowed us to test effectiveness by examining differences in children’s BMI z-scores across the schools over time. Additionally, the nature of the study enabled us to enroll schools based on their motivation, which mirrors the real-life context of school promotion initiatives. The lack of randomization could have introduced confounding biases. To address this, we controlled for the outcomes measured at the baseline, including BMI z-score, sex, age, country, and highest educational level, in all analyses. Future studies should aim for larger sample sizes and longer follow-up periods to better capture the potential long-term benefits of schoolyard greening.

Additionally, we did not control for biological maturation, which could influence individual growth trajectories during this developmental stage. Another important limitation was the dropout rate, particularly in the intervention group (36.7% compared to 12.5% in the control group), which may have introduced attrition bias and affected the generalizability of the results.

Additionally, the study did not account for potential confounding factors such as dietary intake and correction of more detailed SEB variables. Incorporating these variables in future analyses would provide a more detailed understanding of the intervention’s effects.

## 5. Conclusions

The findings of this study suggest that greening schoolyards may have a positive impact on children’s BMI, potentially comparable to those shown by the HPSF interventions, though the effect sizes were small. These findings underscore the possible importance of holistic approaches in tackling childhood obesity effectively. Further research with more school recruited and with an extended follow-up is essential to validate these initial findings and optimize intervention strategies.

## Figures and Tables

**Table 1:** Baseline characteristics at baseline.

	Total (n = 159)		GHPSF (n = 80)		Control (n = 79)		
	N	%/Mean (SD)	N	%/Mean (SD)	N	%/Mean (SD)	*p* Value
Sex	159		80		79		0.817
Boy (%)	71	45	35	44	36	46	
Girl (%)	88	55	45	56	43	54	
Age in years	158	10.2 (1.1)	80	10.1 (1.0)	78	10.2 (1.1)	0.317
Height in cm	157	143.4 (8.3)	80	142.7 (8.0)	77	144.0 (8.5)	0.331
Weight in kg	156	37.6 (8.6)	79	36.3 (8.1)	77	38.4 (9.0)	0.119
WC in cm	157	63.3 (8.4)	80	62.4 (8.3)	77	64.2 (8.5)	0.189
HC in cm	157	77.3 (7.8)	80	76.3 (7.7)	77	78.3 (7.9)	0.124
WHR	157	0.82 (0.05)	80	0.82 (0.05)	77	0.82 (0.05)	0.773
BMI z-score	156	0.41 (1.15)	79	0.29 (1.16)	77	0.52 (1.14)	0.228
IOTF	156		79		77		0.409
Normal weight	119	76	63	80	56	73	
Overweight	26	17	10	13	16	21	
Obese	11	7	6	8	5	6	
SEB ^(a)^	133		66		67		0.987
Low (%)	22	16	11	17	11	16	
Middle (%)	29	22	14	21	15	22	
High (%)	82	62	41	62	41	62	

^(a)^ Socioeconomic background is based on the highest completed education in the family (low = primary school; middle = secondary school; high = higher education/university). GHPSF = Green Healthy Primary School of the Future; WC = waist circumference; HC = hip circumference; WHR = waist hip ratio; BMI = body mass index; IOTF = International Obesity Task Force; SEB = socioeconomic background.

**Table 2:** Estimated intervention effects on anthropometric outcomes at 12 and 18 months: means, standardized effect sizes, and p-values.

Variable	Time	Intervention N; Mean (SD)	Control N; Mean (SD)	Intervention Effect ^(a)^ Difference in Estimated Means (95%CI), *p*-Value	Standardized Effect Size (ES) ^(b)^
BMI z-score	Baseline	79; 0.29 (1.16)	77; 0.52 (1.14)		
	12 months	70; 0.18 (1.23)	50; 0.44 (1.04)	−0.17 (−0.36, 0.02), 0.084	−0.15
	18 months	68; 0.22 (1.19)	51; 0.38 (1.05)	−0.06 (−0.24, 0.13), 0.540	−0.05
Waist circumference	Baseline	80; 62.39 (8.32)	77; 64.16 (8.48)		
	12 months	70; 64.60 (9.03)	50; 65.53 (8.98)	−1.98 (−4.01, 0.06), 0.057	−0.23
	18 months	68; 65.48 (9.43)	51; 66.24 (9.14)	−1.15 (−3.07, 0.76), 0.235	−0.13
Hip circumference	Baseline	80; 76.34 (7.74)	77; 78.28 (7.92)		
	12 months	70; 79.02 (8.75)	50; 77.76 (7.77)	0.45 (−1.10, 2.00), 0.567	0.06
	18 months	68; 79.35 (7.67)	51; 79.56 (8.59)	0.03 (−1.80, 1.87), 0.970	0.005
Waist hip ratio	Baseline	80; 0.82 (0.05)	77; 0.82 (0.05)		
	12 months	70; 0.82 (0.06)	50; 0.84 (0.08)	−0.02 (−0.05, 0.004), 0.090	−0.37
	18 months	68; 0.82 (0.08)	51; 0.83 (0.08)	−0.01 (−0.04, 0.02), 0.420	−0.17

^(a)^ Overall intervention effect: F_2,104.5_ = 2.103 and *p* = 0.127 for BMIz-score; F_2,101.0_ = 1.906 and *p* = 0.154 for waist circumference; F_2,102.9_ = 0.223 and *p* = 0.801 for hip circumference; F_2,100.2_ = 1.505 and *p* = 0.227 for waist hip ratio. All intervention effects (at 12 and 18 months) were corrected for baseline differences and potential confounders (sex, age, country (Belgium/Netherlands), highest education level). ^(b)^ Standardized effect size = intervention effect divided by residual standard deviation of baseline score.

## Data Availability

The original contributions presented in this study are included in the article/Supplementary Materials. Further inquiries can be directed to the corresponding author.

## Supplementary Materials

The following supporting information can be downloaded at https://www.mdpi.com/article/10.3390/children12070944/s1: Table S1. Comparison of standardized effect sizes on BMI z-Score and waist cir-cumference between GHPSF and HPSF at 12 and 48 Months. Table S2a. Stratified Intervention Effects on Anthropometric Outcomes in Boys: Estimated Means, Standardized Effect Sizes, and p-values at Baseline, 12 Months, and 18 Months. Table S2b. Stratified Intervention Effects on Anthropometric Outcomes in Girls: Estimated Means, Standardized Effect Sizes, and p-values at Baseline, 12 Months, and 18 Months.

## Abbreviations

The following abbreviations are used in this manuscript:

BMI	Body Mass Index
ES	Effect Size
GHPSF	Green Healthy Primary School of the Future
HC	Hip Circumference
HPSF	Healthy Primary School of the Future
MAR	Missing at Random
SD	Standard Deviation
SEB	Socioeconomic Background
WC	Waist Circumference
WHR	Waist to Hip Ratio
