# Supplementary material for: Evaluating the Effectiveness of Biodiverse Green Schoolyards on Child BMI z-Score and Physical Metrics: A Pilot Quasi-Experimental Study
Source: Children (Basel). 2025 Jul 17;12(7):944. doi: 10.3390/children12070944 (PMC12293392; doi:10.3390/children12070944)
Supplement: Supplementary file 1 [file children-12-00944-s001.zip › children-3710934-supplementary.pdf]

children

MDPI

Supplementary Materials

Table S1. Comparison of standardized effect sizes on BMI z-Score and waist circumference between GHPSF and HPSF at 12 and 48 Months.

Variable	Time	Standardized effect size (ES) GHPSF	Standardized effect size (ES) HPSF
BMI z-score	12 months	-0.15	-0.04
	48 months		-0.17
Waist circumference	12 months	-0.23	-0.06
	48 months		-0.22

Abbreviations: GHPSF = Green Healthy Primary School of the Future, & HPSF: Healty Primary School of the Future.

Table S2a. Stratified Intervention Effects on Anthropometric Outcomes in Boys: Estimated Means, Standardized Effect Sizes, and p-values at Baseline, 12 Months, and 18 Months.

Variable	Time	Intervention N; mean (SD)	Control N; mean (SD)	Intervention effect [a)] Difference in estimated means (95%CI), p-value	Standardized effect size (ES) [b)]
BMI z- score	Baseline	35; 0.42 (1.17)	36; 0.36 (1.19)		
	12 months	33; 0.15 (1.29)	22; 0.45 (1.18)	-0.26 (-0.65, 0.12), 0.174	-0.20
	18 months	33; 0.11 (1.12)	22; 0.36 (1.20)	-0.11 (-0.44, 0.22), 0.494	-0.08
Waist circumference	Baseline	35; 61.25 (9.53)	36; 64.23 (9.55)		
	12 months	33; 63.73 (9.26)	22; 66.26 (10.64)	-1.49 (-4.23, 1.26), 0.281	-0.15
	18 months	33; 65.03 (10.79)	22; 67.41 (10.50)	-0.55 (-3.30, 2.20), 0.690	-0.05
Hip circumference	Baseline	35; 74.85 (8.50)	36; 78.15 (7.92)		
	12 months	33; 76.42 (8.57)	22; 78.07 (9.77)	-0.28 (-2.91, 2.35), 0.830	-0.03
	18 months	33; 76.58 (7.70)	22; 79.70 (8.50)	-1.75 (-4.54, 1.03), 0.211	-0.21
Waist hip ratio	Baseline	35; 0.82 (0.05)	36; 0.82 (0.05)		
	12 months	33; 0.83 (0.06)	22; 0.85 (0.09)	-0.01 (-0.05, 0.02), 0.476	-0.20
	18 months	33; 0.85 (0.08)	22; 0.84 (0.07)	0.01 (-0.03, 0.05), 0.598	0.14

a) Overall intervention effect: $F_{2,48.2} = 1.172$, p = 0.318 for BMIz-score; $F_{2, 45.6} = 0.604$, p = 0.551 for waist circumference; $F_{2, 48.8} = 1.016$, p = 0.370 for hip circumference; $F_{2, 44.9} = 0.499$, p=0.611 for waist hip ratio; all intervention effects (at 12 and 18 months) were corrected for baseline differences and potential confounders (sex, age, country (Belgium/Netherlands), highest education level).

b) Standardized effect size = intervention effect divided by residual standard deviation of baseline score.

Table S2b. Stratified Intervention Effects on Anthropometric Outcomes in Girls: Estimated Means, Standardized Effect Sizes, and p-values at Baseline, 12 Months, and 18 Months.

Variable	Time	Intervention N; mean (SD)	Control N; mean (SD)	Intervention effect [a)] Difference in estimated means (95%CI), p-value	Standardized effect size (ES) [b)]
BMI z- score	Baseline	44; 0.19 (1.16)	41; 0.65 (1.09)		
	12 months	37; 0.21 (1.20)	28; 0.43 (0.93)	-0.08 (-0.24, 0.07), 0.266	-0.08
	18 months	35; 0.33 (1.27)	29; 0.39 (0.95)	0.001 (-0.21, 0.21), 0.994	0.001
Waist circumference	Baseline	45; 63.29 (7.23)	41; 64.10 (7.55)		
	12 months	37; 65.38 (8.87)	28; 64.96 (7.59)	-2.42 (-5.43, 0.59), 0.113	-0.33
	18 months	35; 65.90 (8.08)	29; 65.35 (8.03)	-1.85 (-4.60, 0.90), 0.183	-0.25
Hip circumference	Baseline	45; 77.50 (6.98)	41; 78.39 (8.02)		
	12 months	37; 81.33 (8.35)	28; 77.51 (5.94)	1.20 (-0.66, 3.06), 0.202	0.17
	18 months	68; 82.36 (8.53)	51; 79.09 (7.11)	1.40 (-1.06, 3.85), 0.259	0.20
Waist hip ratio	Baseline	45; 0.82 (0.06)	41; 0.82 (0.05)		
	12 months	37; 0.80 (0.06)	28; 0.84 (0.07)	-0.04 (-0.08, 0.003), 0.067	-0.62
	18 months	35; 0.80 (0.06)	29; 0.83 (0.08)	-0.03 (-0.07, 0.01), 0.058	-0.58

a) Overall intervention effect: $F_{2,54.9} = 1.170$, p = 0.318 for BMIz-score; $F_{2, 52.9} = 1.528$, p = 0.226 for waist circumference; $F_{2, 51.2} = 0.990$, p = 0.379 for hip circumference; $F_{2,55.0} = 2.764$, p=0.072 for waist hip ratio; all intervention effects (at 12 and 18 months) were corrected for baseline differences and potential confounders (sex, age, country (Belgium/Netherlands), highest education level).

b) Standardized effect size = intervention effect divided by residual standard deviation of baseline score.
